# Supplementary material for: Mixotrophy for carbon-conserving waste upcycling
Source: PLoS Comput Biol. 2025 Aug 26;21(8):e1013379. doi: 10.1371/journal.pcbi.1013379 (PMC12407552; doi:10.1371/journal.pcbi.1013379)
Supplement: Attachment — Submitted filename: ReviewComments_Mixotrophy_Cnecator.pdf [file pcbi.1013379.s008.pdf]

Review comments for

Mixotrophy for carbon-conserving waste upcycling

Submitted by Michael Weldon and Christian Euler to *PLoS Computational Biology*

The presented work revolves around the computational analysis of the metabolism of *Cupriavidus necator*, a soil-bacterium gaining popularity in academic and industrial research due to its natural ability to produce the bioplastic polymer PHB as well as its versatile metabolism. The latter includes the ability for chemolithoautotrophic growth on CO_2 via the Calvin-Benson-Bassham (CBB) cycle fuelled by H_2 and O_2, giving *C. necator* the nickname of a "Knallgas" bacterium. This metabolic versatility is at the core of the manuscript by Weldon and Euler, as they simulate *C. necator*'s ability to individually utilise different compounds derived from waste streams as substrates for growth or production of PHB. In most cases, the respiratory metabolism employed by *C. necator* for these processes would result in the release of CO_2, which in theory could be avoided if the cell is given enough additional reducing power to run the CBB cycle and reassimilate this CO_2. The authors use a model of the core metabolism of *C. necator* to analyse several scenarios in which this additional reducing power is provided by different forms of mixotrophy, either in the form of additional H_2 or co-utilisation of one of the other compounds derived from waste streams, and finally only partial oxidation of some of the compounds. Their list of candidate compounds covers thereby a range of reducing potentials, allowing for a broader analysis of possible scenarios.

The study focuses on stoichiometry as well as thermodynamic feasibility of the different mixotrophy scenarios, whereby the size and coverage of the metabolic model is reasonable and the employed methods for simulating relative fluxes and thermodynamic driving forces are widely established and appropriate. The model files as well as the code to reproduce their analyses and figures is readily available online.

Generally, the work addresses a current and relevant challenge, namely the efficient re-utilisation of waste products to move towards a circular carbon economy, using biotechnology as one means to aid in this process. The focus on H_2-utilisation and reduction in CO_2-emissions in this context aligns furthermore well with current trends in industry and governmental policies.

While microbial utilisation of waste products has been the subject of experimental and computational research in the past (including co-utilisation with the addition of H_2), the systematic analysis of exemplary mixotrophic scenarios in *C. necator* presents a novelty useful to the field, providing in-silico insights in the potential efficiency of different co-utilisation scenarios that can guide researchers and engineers in developing bioprocesses revolving around CO_2-neutral waste product utilisation employing *C. necator*.

The acknowledgement of the university's location on traditional land of indigenous peoples is furthermore appreciated.

Despite the overall positive impression, there are several points I believe will raise clarity and/or quality of the presented research:

Major revision points:

Major 1: The authors claim no CO_2 reassimilation is necessary to produce PHB from acetate and EtOH (line 128), resulting in the missing bar/asterisk in Figure 1C for both compounds. First, I believe the sentence (line 128-129) is missing the crucial part of "for full carbon conversion", as also all other compounds can produce PHB without CO_2 reassimilation (with exception of CO_2, of course), if full carbon conversion is neglected. Second, while I agree with the conclusion for EtOH as its assimilation into acetyl-CoA provides the necessary reducing power to go from acetyl-CoA to PHB (Figure 2C), this is not true for acetate, as you state yourself in the subsequent sentence (line 129-130) and even show in Figure 2D. It remains true that there might be no flux through RuBisCO in the "Reassimilation Forced" scenario (Figure 2D, lower), but there is still a requirement for H_2 to make this scenario *carbon neutral*, which renders the results for acetate described, and shown in Figure 1D, confusing if not misleading. Yes, there is no H_2 cost for CO_2 reassimilation in the acetate scenario, but there is a H_2 cost for not producing CO_2 (=full carbon conversion). I suggest relabelling this analysis as "H_2 requirement for zero net CO_2-production". This should leave all other results in Figure 1D unchanged, including EtOH, but acetate will now have its correct H_2 cost associated with its full carbon conversion. Similar for Figure 1E, which will have acetate added (EtOH should still be left out or could be sitting on the X-axis). Subsequent statements and interpretations in the text regarding acetate will have to be adjusted accordingly, if necessary.

Major 2: In the Carbon-carbon mixotrophy section (lines 203-268) the authors claim that the mixotrophic scenario must have a similar or higher MDF than the compounds individually to be viable, and the subsequent interpretation of the results builds on this hypothesis. The authors should provide evidence/references that this statement/hypothesis is true. As long as one of the carbon substrates does not render the assimilation of the other one thermodynamically infeasible (dG>0), why would it not be taken up and metabolised? (Only accounting for stoichiometry + thermodynamics of course, not regarding allosteric regulations, catabolite repression, etc which are not part of the model/analysis).

Major 3: I did not find any constraints set on the ratios of ATP/ADP, NADH/NAD or NADPH/NADP, at least not especially mentioned. Optimisation algorithms like MDF have the tendency to go for extremes in certain boundaries (in the case of MDF the

metabolite concentrations). As the authors don't go into detail as to which reactions are causing the thermodynamic limits (=operate at the MDF) for each case but rather report the MDF value for the whole scenario, it could be that unphysiological extremes in the ratios of these co-factor pairs occur, leading to distorted MDF values influencing the overall results. Especially with the focus in this study on availability of reducing power, I believe an explorative analysis of these ratios for the different scenarios is appropriate as a first step (including a comparison with reported values from literature, even if not *C. necator*). Depending on the outcome additional constraints on the ratios might be required. From briefly skimming the optMDFPathway description, it allows setting individual values and/or ranges for these ratios.

Major 4: While I do appreciate this being a purely computational study, there exist recent published experimental data with high relevance to the presented work, e.g. https://doi.org/10.3390/fermentation8030125. I highly suggest a more intense literature search to find relevant experimental data for validation of the modelling results, if only so in trends or tendencies.

Minor revision points:

Minor 1: Excluding transport reactions from the thermodynamic analysis for simplicity reasons is understandable, although the transport of some compounds, especially charged ones, can influence the thermodynamic landscape of the network. Some carbon source uptake might require active transport coupled to ATP hydrolysis, for example. I suggest rephrasing that statement (or include the thermodynamics of transport reactions, assuming a reasonably high external concentration).

Minor 2: A more recent source for fossil fuels running out at the end of the century than 2010 (ref 3, Stephens et al.) would be appropriate. Global economies and associated fossil resource usage changed *somewhat* over the last 15 years.

Minor 3: The manuscript text mentions Figure 1E to be about PHB production, which is not mentioned in the Figure description

Minor 4: The claim of creating 90 models (lines 208-209), but only 55 (45 + 10 original) shown in Figure 3A. Were there really 90 created or rather 45 due to double matching of compounds creating identical models twice? The number 90 is confusing in this context.

Minor 5: The labelling in Figure S1 is switched. "w/Reassim." shows lower yields, contradicting statements in the text.

Formatting/Typos:

- Wood-Ljungd**ah**l (line 35)
- Close bracket for Y-axis label in Figure 1C and 1E
- Usage of *hydrogen* (written out) vs H_2 throughout the text
- Usage of abbreviation DOR vs Degree of Reduction in Figure 1E
