# Supplementary material for: Mixotrophy for carbon-conserving waste upcycling
Source: PLoS Comput Biol. 2025 Aug 26;21(8):e1013379. doi: 10.1371/journal.pcbi.1013379 (PMC12407552; doi:10.1371/journal.pcbi.1013379)
Supplement: Attachment — Submitted filename: Mixotrophy_resubmission_letter.pdf [file pcbi.1013379.s009.pdf]

Dr. Christian Euler
Dept. of Chemical Engineering
University of Waterloo
200 University Ave., E6-4020
Waterloo, ON, N2L 3G1

To Drs. Feilim Mac Gabhann & Jason Papin
Editors-in-Chief
PLOS Computational Biology

June 5, 2025

Dear Drs. Feilim Mac Gabhann & Jason Papin

Please accept our revised original research article entitled "Mixotrophy for carbon-conserving waste upcycling" for consideration for publication in *PLOS Computational Biology*. This submission is original and has not been considered for publication elsewhere. A pre-print has been shared on the bioRxiv under the same title.

We thank the reviewers for their constructive feedback on our initial manuscript submission. We have addressed all points, and we hope that our edits have satisfied the reviewers' critiques. Please find our itemized response to reviewer feedback attached to this letter.

Sincerely,

Dr. Christian Euler
ceuler@uwaterloo.ca
Assistant Professor
Department of Chemical Engineering
University of Waterloo

Major 1: The authors claim no CO2 reassimilation is necessary to produce PHB from acetate and EtOH (line 128), resulting in the missing bar/asterisk in Figure 1C for both compounds. First, I believe the sentence (line 128-129) is missing the crucial part of "for full carbon conversion", as also all other compounds can produce PHB without CO2 reassimilation (with exception of CO2, of course), if full carbon conversion is neglected. Second, while I agree with the conclusion for EtOH as its assimilation into acetyl-CoA provides the necessary reducing power to go from acetyl-CoA to PHB (Figure 2C), this is not true for acetate, as you state yourself in the subsequent sentence (line 129-130) and even show in Figure 2D. It remains true that there might be no flux through RuBisCO in the "Reassimilation Forced" scenario (Figure 2D, lower), but there is still a requirement for H2 to make this scenario carbon neutral, which renders the results for acetate described, and shown in Figure 1D, confusing if not misleading. Yes, there is no H2 cost for CO2 reassimilation in the acetate scenario, but there is a H2 cost for not producing CO2 (=full carbon conversion). I suggest relabelling this analysis as "H2 requirement for zero net CO2-production". This should leave all other results in Figure 1D unchanged, including EtOH, but acetate will now have its correct H2 cost associated with its full carbon conversion. Similar for Figure 1E, which will have acetate added (EtOH should still be left out or could be sitting on the X-axis). Subsequent statements and interpretations in the text regarding acetate will have to be adjusted accordingly, if necessary.

We appreciate the reviewer's point and have made all suggested changes. We also elected to characterize the hydrogen cost on a carbon-mole basis based on this comment, as we think this is the clearest comparison between carbon sources. This resulted in a new figure (Fig. S2) showing hydrogen cost reductions relative to C1 transformation.

Major 2: In the Carbon-carbon mixotrophy section (lines 203-268) the authors claim that the mixotrophic scenario must have a similar or higher MDF than the compounds individually to be viable, and the subsequent interpretation of the results builds on this hypothesis. The authors should provide evidence/references that this statement/hypothesis is true. As long as one of the carbon substrates does not render the assimilation of the other one thermodynamically infeasible (dG¿0), why would it not be taken up and metabolised? (Only accounting for stoichiometry + thermodynamics of course, not regarding allosteric regulations, catabolite repression, etc which are not part of the model/analysis).

We have changed the language of this specific section to reflect the fact that this is our novel argument and to provide some more justification for it. We have also provided citations to support the idea that MDF is a measure of relative pathway performance, which helps to justify our position.

Major 3: I did not find any constraints set on the ratios of ATP/ADP, NADH/NAD or NADPH/NADP, at least not especially mentioned. Optimisation algorithms like MDF have the tendency to go for extremes in certain boundaries (in the case of MDF the metabolite concentrations). As the authors don't go into detail as to which reactions are causing the thermodynamic limits (=operate at the MDF) for each case but rather report the MDF value for the whole scenario, it could be that unphysiological extremes in the ratios of these cofactor pairs occur, leading to distorted MDF values influencing the overall results. Especially with the focus in this study on availability of reducing power, I believe an explorative analysis

of these ratios for the different scenarios is appropriate as a first step (including a comparison with reported values from literature, even if not C. necator). Depending on the outcome additional constraints on the ratios might be required. From briefly skimming the optMDF-Pathway description, it allows setting individual values and/or ranges for these ratios.

To the best of our knowledge, at the time of writing of the initial draft there were no reports of reasonable cofactor ratios for *C. necator*, so we initially performed our analysis using highly permissive concentration bounds as in [1]. However, measured cofactor ratios for *C. necator* in mixotrophic scenarios were recently published [2], so we have updated our analysis using these bounds instead. This significantly changed the specific results of our analysis, but did not significantly change the overall outcomes. We have added language making the bounds we used explicit and have included a new file with cofactor bounds in the Github repository. We have also highlighted the reactions operating at the MDF for each flux distribution presented and summarized the overall trend we observed in terms of thermodynamic bottlenecks across the carbon sources we investigated.

Major 4: While I do appreciate this being a purely computational study, there exist recent published experimental data with high relevance to the presented work, e.g. https://doi.org/10.3390/fermentation8030125. I highly suggest a more intense literature search to find relevant experimental data for validation of the modelling results, if only so in trends or tendencies.

We thank the reviewer for the reference suggestion and have included it in our literature review. We additionally included [2] because it is highly relevant to the work.

Minor 1: Excluding transport reactions from the thermodynamic analysis for simplicity reasons is understandable, although the transport of some compounds, especially charged ones, can influence the thermodynamic landscape of the network. Some carbon source uptake might require active transport coupled to ATP hydrolysis, for example. I suggest rephrasing that statement (or include the thermodynamics of transport reactions, assuming a reasonably high external concentration).

We agree with the reviewer that uptake reactions might change some of the results we have generated. However, it is unclear which specific language the reviewer would like us to address. As such, our justification for excluding transport reactions remains unchanged.

Minor 2: A more recent source for fossil fuels running out at the end of the century than 2010 (ref 3, Stephens et al.) would be appropriate. Global economies and associated fossil resource usage changed somewhat over the last 15 years.

We have updated the manuscript to include two more recent references. Unfortunately, it appears as though the change in usage has been for the negative, and fossil fuels are expected to run out faster than in the original 2010 reference we included.

Minor 3: The manuscript text mentions Figure 1E to be about PHB production, which is not mentioned in the Figure description

We have addressed this issue.

Minor 4: The claim of creating 90 models (lines 208-209), but only 55 (45 + 10 original) shown in Figure 3A. Were there really 90 created or rather 45 due to double matching of compounds creating identical models twice? The number 90 is confusing in this context.

The reviewer is correct and the number of models should only be 45 in this sentence. It has been corrected as such.

Minor 5: The labelling in Figure S1 is switched. "w/Reassim." shows lower yields, contradicting statements in the text.

This has been corrected.

References

[1] M. Janasch, N. Crang, J. Asplund-Samuelsson, E. Sporre, M. Bruch, A. Gynnå, M. Jahn, and E. P. Hudson, "Thermodynamic limitations of phb production from formate and fructose in cupriavidus necator," *Metabolic Engineering*, vol. 73, pp. 256–269, 9 2022.

[2] J. Feng, X. Li, X. Teng, D. Fan, J. Yin, Y. Qiu, Z. Yi, L. Chen, H. M. Zhang, and C. Rao, "Harnessing co2 fixation and reducing power recycling for enhanced polyhydroxyalkanoates industrial bioproduction," *Metabolic Engineering*, vol. 91, pp. 204–216, 9 2025.
